# Sputum-Septicæmia

**Published:** 1884-06

**Authors:** W. D. Miller

**Affiliations:** Berlin, Germany


### SPUTUM-SEPTICÆMIA.

BY W. D. MILLER, BERLIN, GERMANY.

At a recent sitting of the *Congress fur Innere Medecin*, Dr. A. Fraenkel gave an account of a form of Septicæmia produced by inoculating rabbits with the sputum of healthy individuals. Just before the death of the animal, which occurred about twenty-four hours after inoculation, the fungus appeared in the blood in large numbers, in the form of cocci and diplococci.

This discovery is interesting in the first place, from the fact that the cocci of sputum-septicæmia are surrounded by a gelatinous sheath, identical with that which characterizes the cocci of pneumonia, and which was thought to be peculiar to the latter.

It is interesting in the second place, because it shows in the human mouth the presence of an element of disease not yet recognized. Whether this fungus ever occurs in the human mouth in sufficient numbers to render possible a case of blood poisoning from one's own saliva, is a question which cannot at present be answered.

The mouth, placed at the very portal of the human body, appears to serve as an incubator for the most various germs of disease, and it is highly probable that the importance of proper cleanliness from this point of view has not been thoroughly appreciated.

Even in the question of caries of the teeth, it is not at all likely that all the parasitic factors have been ferreted out.

In my last experiments I have succeeded in finding and isolating other acid-producing organisms, besides those described in the

May number of the INDEPENDENT PRACTITIONER; organisms which, as far as the microscope reveals, are identical with the latter, but which are entirely different physiologically. We have in the oral cavity a field of labor that, to all appearances, will not be exhausted for years to come.

At the same meeting were also demonstrated preparations of the fungus of pneumonia. This fungus occurs regularly in forms of cocci and diplococci; in some cases, however, bacilli are also produced, and not infrequently a coccus is seen united to a bacillus, thus confirming what I have already demonstrated for various fungi the fact that a given fungus may produce different forms of development, such as cocci, bacilli, bacteria, etc.

